# The crown-root morphology of central incisors in different skeletal malocclusions assessed with cone-beam computed tomography

**DOI:** 10.1186/s40510-019-0272-2

**Published:** 2019-05-21

**Authors:** Xiao-ming Wang, Ling-zhi Ma, Jing Wang, Hui Xue

**Affiliations:** 1 State Key Laboratory of Oral Diseases, Department of Cleft Lip and Palate Surgery, West China Hospital of Stomatology, Sichuan University, Chengdu, 610041 China; 2 Department of Orthodontics, Stomatological Hospital of Kunming Medical University, Kunming, 650032 China; 3 Department of Orthodontics, Xi’an JiaoTong University Hospital of Stomatology, Xi’an, 710004 Shaanxi Province China; 4 Department of Stomatology, The Affiliated Suzhou Hospital of Nanjing Medical University, Suzhou, 215000 Jiangsu Province China

**Keywords:** Crown-root morphology, Skeletal malocclusion, Collum angle, Labial surface angle, Cone-beam CT

## Abstract

**Background:**

To determine the discrepancy of crown-root morphology of central incisors among different types of skeletal malocclusion using cone-beam computed tomography (CBCT) and to provide guidance for proper torque expression of anterior teeth and prevention of alveolar fenestration and dehiscence.

**Methods:**

In this retrospective study, a total of 108 CBCT images were obtained (ranging from 18.0 to 30.0 years, mean age 25.8 years). Patients were grouped according to routine sagittal and vertical skeletal malocclusion classification criteria. The patients in sagittal groups were all average vertical patterns, with Class I comprised 24 patients—14 females and 10 males; Class II comprised 20 patients—13 females and 7 males; and Class III comprised 22 subjects—13 females and 9 males. The patients in vertical groups were all skeletal Class I malocclusions, with low angle comprised 21 patients—12 females and 9 males; average angle comprised 24 patients; and high angle comprised 21 patients—11 females and 10 males. All the CBCT data were imported into Invivo 5.4 software to obtain a middle labio-lingual section of right central incisors. Auto CAD 2007 software was applied to measure the crown-root angulation (Collum angle), and the angle formed by a tangent to the central of the labial surface of the crown and the long axis of the crown (labial surface angle). One-way analysis of variance (ANOVA) and Scheffe’s test were used for statistical comparisons at the *P* < 0.05 level, and the Pearson correlation analysis was applied to investigate the association between the two measurements.

**Results:**

The values of Collum angle and labial surface angle in maxillary incisor of Class II and mandibular incisor of Class III were significantly greater than other types of sagittal skeletal malocclusions (*P* < 0.05); no significant difference was detected among vertical skeletal malocclusions. Notably, there was also a significant positive correlation between the two measurements.

**Conclusions:**

The maxillary incisor in patients with sagittal skeletal Class II malocclusion and mandibular incisor with Class III malocclusion present remarkable crown-root angulation and correspondingly considerable labial surface curvature. Equivalent deviation during bracket bonding may cause greater torque expression error and increase the risk of alveolar fenestration and dehiscence.

## Backgrounds

**Fig. 1:**
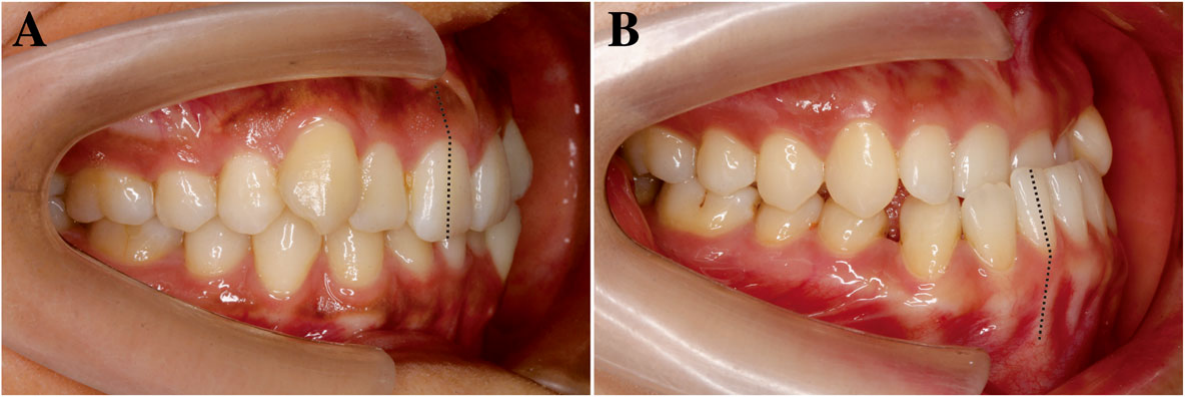
**a**, **b** The inclination of the root and crown in maxillary and mandibular incisors are inconsistent with each other in the surface view, which indicates the crown-root angulation phenomenon

Adequate labial or lingual inclination of anterior teeth is important to establish the ideal anterior occlusal relationship and satisfying esthetic effect in orthodontics. However, orthodontists cannot always achieve the expected extent of tooth movement in alveolar bone. Researchers paid plenty of attention to the alveolar height and thickness in the past two decades, while the tooth morphological variation was frequently ignored (Fig. 1). In 1984, Bryant firstly analyzed the variability in the permanent incisor morphology by establishing three anatomic features and investigated the discrepancy among different malocclusions [1], two of which adopted by the following studies [2].

One feature was the crown-root angulation (Collum angle, CA) in a labiolingual direction, which was formed by the long axis of crown and root and might limit the degree to which the roots of incisor could be torqued lingually for relating to the lingual cortical plate of bone. Later, several recent studies suggested that the CA caused abnormal stress distribution of periodontal ligament when tooth movement [3, 4]. Moreover, researchers found the mean value of CA for Angle Class II division 2 malocclusion was significantly larger than Class II division 1 and Class III malocclusions [4–10]. The above research implied us to furtherly investigate the diversity among different skeletal malocclusions.

The other feature was the labial surface angle (LSA), which was formed by a tangent to the bracket site on the labial surface and the long axis of the crown from a proximal view, and the significant amount of variation in LSA potentially affected the precision of torque expression and axial inclination [2]. Kong drew a tangent to the labial surface of the crown 3.5–5.0 mm gingivally from the incisal edge and measured the LSA of 77 incisors [2]. He demonstrated that the significant variation in LSA was greater than the variations between different types of preadjusted appliances, and the brackets still needed to be custom-made when using the straight-wire approach [2]. Thus, the preoperative judgment about the individual LSA was essential for achieving optimal torque expression. Moreover, the developmental tooth proved to be closely affected by environmental and genetic factors, which seemed coincident with the determinants of the facial growth pattern, while little was known about the correlation to the different skeletal malocclusions [11, 12].

Previous research primarily based on cephalometric radiographs and the disadvantages of magnifying distortion and unclear manual tracing of the tooth boundary might sacrifice the accuracy. Currently, CBCT is widely used in the clinic with abundant sample sources, clear three-dimensional imaging of tooth bone structure, and precise measurement via digital software, but the application in tooth morphometry remains rare [13–15].

The main purposes of the present study were to investigate the variations in the morphology of maxillary and mandibular central incisors, including the CA and LSA, using CBCT images and Invivo 5.4 software to capture images, and analyzing via AutoCAD. Finally, we discussed the effect on torque expression for the variable anatomic feature among different types of skeletal malocclusions.

## Material and methods

### Trial design

Firstly, a power analysis established by G*Power (version 3.1.9.4, Franz Faul, Universita¨t Kiel, Kiel, Germany) software, based on 1:1 ratio between groups, with sample size of 108 cases, would give more than 70% power to detect significant differences with 0.40 effect size and at the *α* = 0.05 significance level.

### Sample selection and classification

The study was carried out on the CBCT scans of three classifications of the sagittal skeletal malocclusions selected from the archives of the Department of Stomatology, the Affiliated Suzhou Hospital of Nanjing Medical University. By August 2018, 2855 sets of images were stored in the database of the department. Because our study was a retrospective case-control study using the archive, no ethical approval was gained, and all the patients took CBCT for clinical orthodontic needs.

The CBCT images of 108 patients (mean age 25.8 years, 18 to 30 years) were selected as the criteria presented in Table 1. CBCT images were obtained using the GALILEOS (SIRONA, Germany), with a visual range of 150 × 150 mm^2^, tube voltage of 90 kV, tube current of 7.0 mA, slice thickness of 0.20 mm, exposure time of 20 s, and radiation dose of 0.029 mSv. During scanning, patients should parallel the interpupillary line and Frankfurt plane to the ground, and the facial midline coincided to the median reference line of the machine, with central occlusion and no swallow.

**Table 1:** Criteria for sample selection

Inclusion criteria	Exclusion criteria
Permanent dentition, completely developed root, no apparent bending and no absorbing	Anterior root with periapical lesions or apparent bending, containing embedded supernumerary teeth in alveolar bone
Intact contour of the crown, and no apparent abrasion	Crown with obvious abrasion
Moderate crowding, and no apparent rotation in anterior teeth	Mild to severe crowding, or obvious rotation in anterior teeth
No caries, filling, restoration history and periodontitis in anterior teeth	Caries, filling or restoration treatment, or periodontitis leading to loosening in anterior teeth
No orthodontic, functional orthopedic treatment, cleft lip palate, and orthognathic surgery history	With orthodontic, functional orthopedic treatment, cleft lip palate, and orthognathic surgery history
No oral bad habit, occlusion interference, swallowing and respiratory disorder, and facial or spinal abnormalities	With oral bad habit and the mandibular located in functional and unstable position, or jaw cyst, cancer, injury, and abnormalities
Clear imaging by CBCT	Blurring image by CBCT

**Fig. 2:**
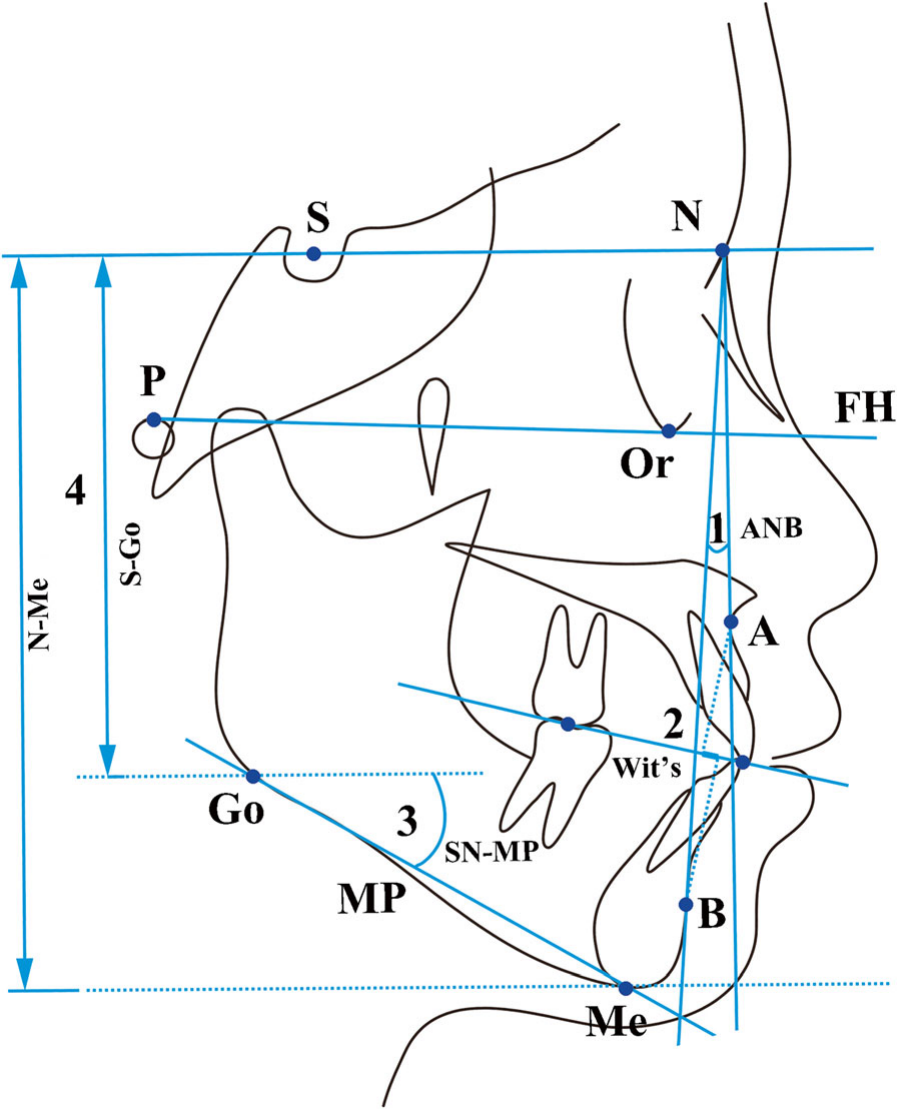
Measurements to classify sagittal and vertical skeletal malocclusion. A, A-point, deepest bony point on the contour of the premaxilla below ANS; B, B-point, deepest bony point on the contour of the mandible above pogonion; ANB, angle between point A, B and point N; 1. Wits, perpendicular lines are dropped from points A and B onto the occlusal plane, Wits is measured from Ao to Bo; 2. S, sella, center of sella turcica; N, nasion, the most anterior limit of the frontonasal suture on the frontal bone in the facial midline; SN, connection between S and N, stands for anterior cranium base plane; Go, gonion, the most posterior inferior point of mandible angle; Me, menton, most inferior point of the bony chin; MP, connection between Me and Go, stands for mandibular plane; SN-MP, angle between SN and MP; 3. S-Go, the distance between lines parallel to FH plane passing through S and Go, represents the posterior facial height; N-Me, the distance between lines parallel to FH plane passing through N and Me, represents for the anterior facial height; FHI(S-Go/N-Me), facial height index, the ratio of posterior and anterior height, stands for vertical growth pattern of individual

Lateral cephalometric radiographs were captured using Invivo 5.4 software and then classified into three groups on the basis of sagittal skeletal malocclusion using Dolphin 11.0 for cephalometric analysis (Fig. 2). The grouping criteria and sample distribution were presented in Table 2 [2, 16, 17].

**Table 2:** The distribution of samples

Skeletal malocclusion		Criteria for sample classification	Number
Class I	Average angle	1° ≤ ANB ≤ 5°, − 3.6 mm ≤ Wits ≤ 0.7 mm	24 (10 male)
Class II		ANB > 5°, Wits > 2 mm	20 (7 male)
Class III		ANB < 1°, Wits < − 3.6 mm	22 (9 male)
Low angle	Class I	SN-MP < 27 °, FHI(S-Go/N-Me) > 68%	21 (9 male)
Average angle		27° ≤ SN-MP ≤ 37° , 62% ≤ FHI(S-Go/N-Me) ≤ 68%	24 (10 male)
High angle		SN-MP > 37°, FHI(S-Go/N-Me) < 62%	21 (10 male)

*CA* Collum angle, *LSA* labial surface angle, *SD* standard deviation, *I* Class I, *II* Class II, *III* Class III, *L* low angle, *A* average angle, *H* high angle

### Measuring image capture

**Fig. 3:**
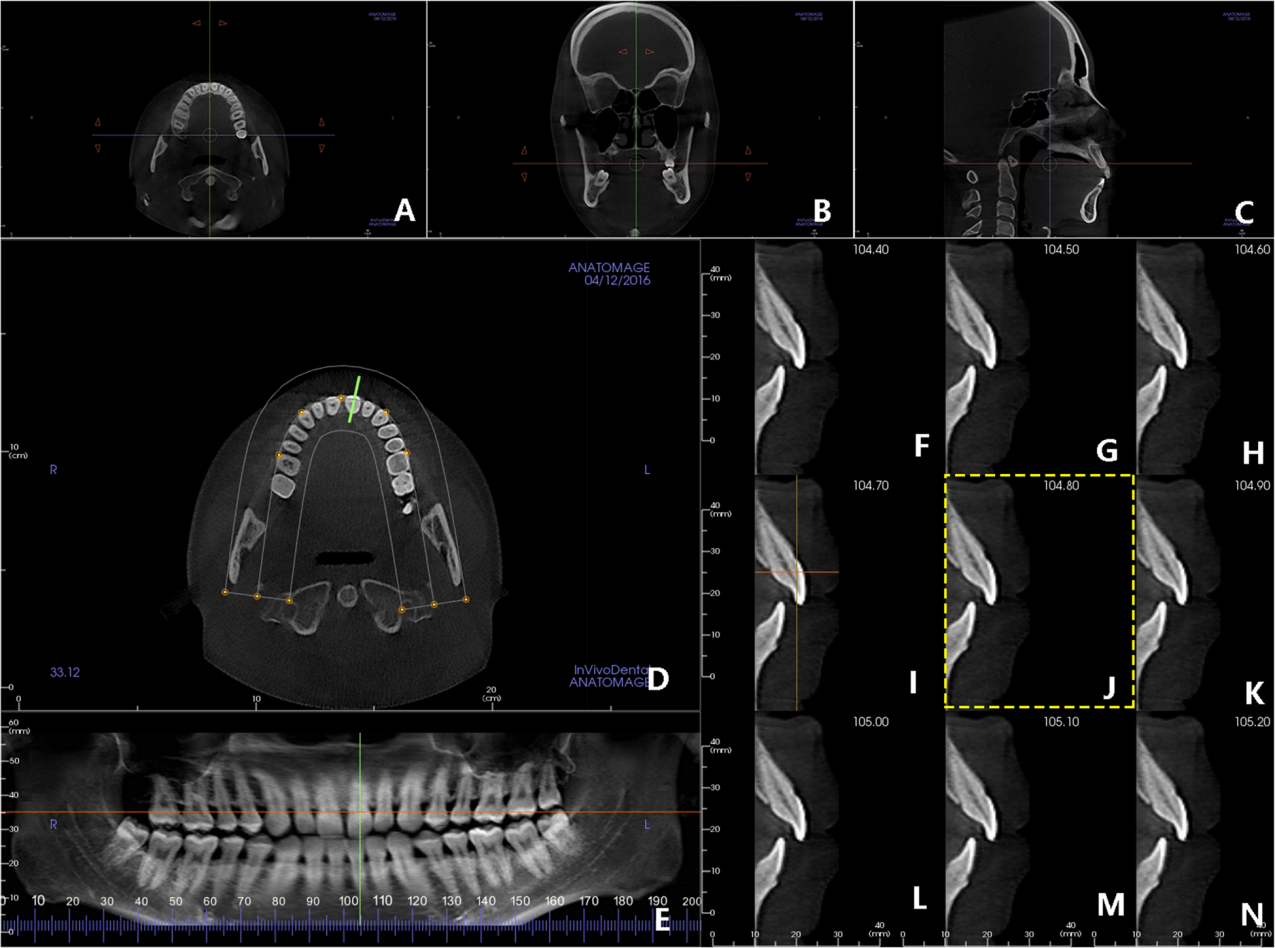
Measuring image capture. The natural position of the head is adjusted in three dimensions. **a** The horizontal view. **b** The coronal view. **c** The sagittal view. A bunch of cutting lines (green) was vertical to incisor labial surface (**d**) and located at the central coronal view (**e**). The median sagittal views were established with nine layers (**f**–**n**), interval 0.10 mm, and the middle one was the measuring image (**j**)

The CBCT images underwent a three-dimensional adjustment with Invivo 5.4 software (Anatomage Dental) to orient the head in natural head position in three planar views. Firstly for the horizontal view, the horizontal line located rightly at the frontal edges of the bilateral ramus, and the vertical line was perpendicular to it and passed through the center of the incisive canal (Fig. 3a). Then for the coronal view, the vertical line should be parallel to the mid-sagittal reference line at crista galli (Fig. 3b). Lastly for the sagittal view, the horizontal line connecting the anterior nasal spine to the posterior nasal spine should be parallel to the bottom of the monitor (Fig. 3c).

Then, the median sagittal tomographic images of incisors in labio-lingual direction were adjusted to capture using the *Arch Section* tab. In detail, the bunch of cutting lines (green) should be vertical to the labial surface and pass through the center in horizontal view (Fig. 3d) and divide incisor equally in coronal view (Fig. 3e). Thus, the median one (Fig. 3j) of the nine images (Fig. 3f–n) in sagittal direction was selected for angular measurement. The thickness of sectional slices was 2.0 mm with the interval set at 0.1 mm.

### Marker and measurement

**Fig. 4:**
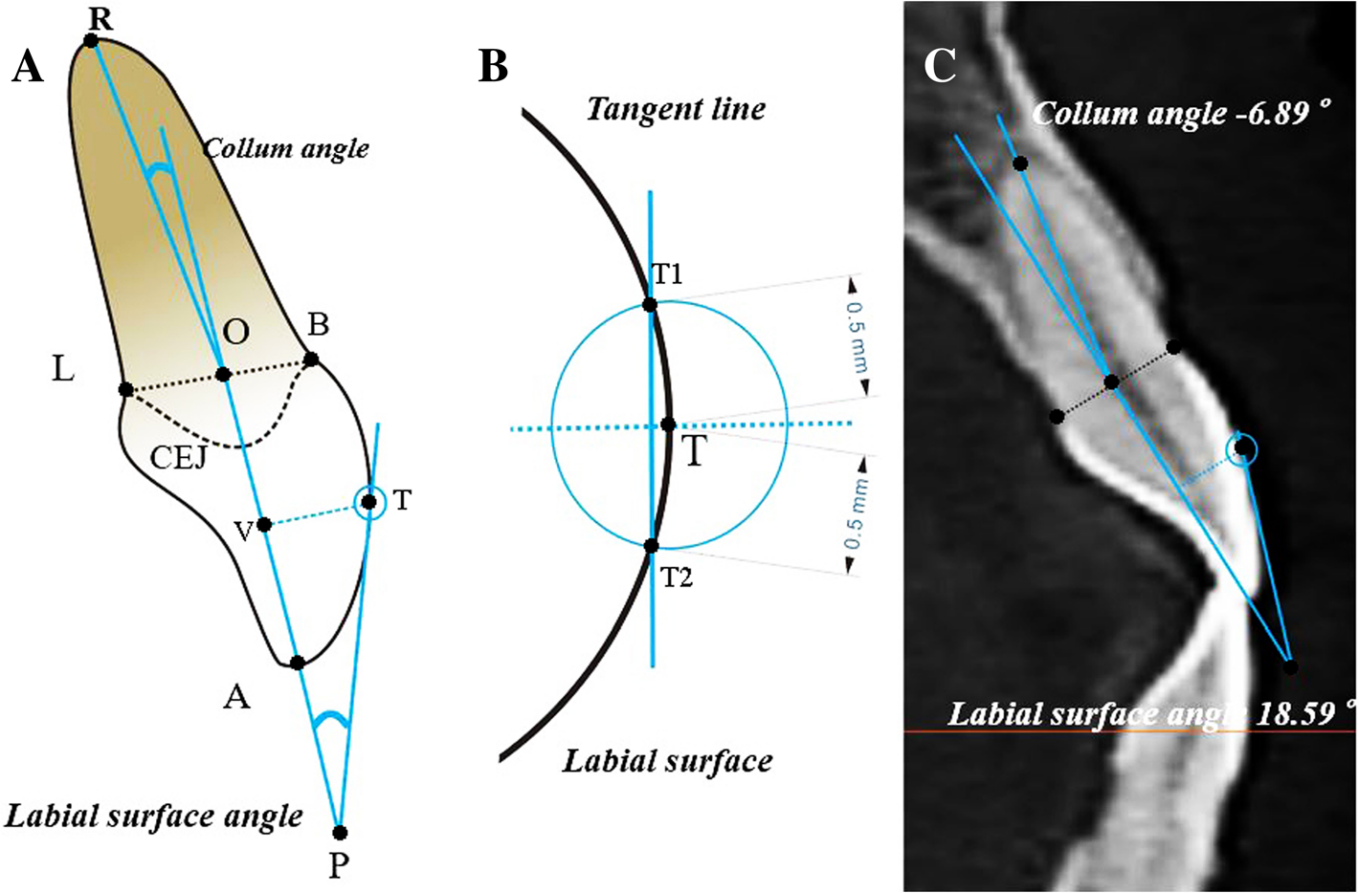
**a** The Collum angle is formed by the extension of the long axis of the crown and the long axis of the root. **b** Tangent L passes through upper and lower intersections of labial surface of crown and circle with the T center and radius of 0.5 mm. **c** The measuring example of Collum angle and labial surface angle

The measuring images were marked and measured via AutoCAD (Autodesk, San Rafael, CA) as follows (Fig. 4a). “CEJ” represented the labial or lingual cementoenamel junction. Point A was the incisor superior, and point R was the root apex. Point B was labial cementoenamel junctions, point L was lingual cementoenamel junctions, and point O was the midpoint between points B and L.

The straight line “AO” represented the long axis of the crown, and “RO” was the long axis of the root. Point T was the tangent point on the labial surface of the crown, which was the intersection of the perpendicular line of “AO” and the labial surface of the crown, with the foot point V. The tangent line via T was defined approximately by the line passing through points T1 and T2, which were the intersections of a circle with the point T center and 0.5 mm radius on the labial surface of the crown (Fig. 4b).

“Collum angle (CA)” was an acute angle between the line RO and reverse extension line AO. When line RO located lingual side to the extension line, the CA was defined as a positive value; otherwise, the labial side was negative, and the coincidence was zero. “Labial surface angle (LSA)” was formed by the tangent line and forward extension line of AO, with point P as the vertex. For example, the CA was − 6.89° and LSA was 18.59° (Fig. 4c).

### Statistical analysis

All statistical analyses were performed with the SPSS software (version 13.0, SPSS, Chicago). The normality test of Kolmogorov-Smirnov and Levene’s variance homogeneity test with all the data were found to be normally distributed with the homogeneity of variance among groups. Further statistical comparisons of CA and LSA in different malocclusion groups were undertaken by one-way analysis of variance (ANOVA) and Scheffe’s test. At last, the Pearson correlation analysis was applied to investigate the association between CA and LSA in the same incisor (“r” was the Pearson correlation coefficient). The level of statistical significance was set at *P* < 0.05(*), *P* < 0.01(**), and *P* < 0.001(***).

### Error in measurements

To assess the intra-observer and inter-observer error, repeated measurements performed on all the samples were measured by two operators on two occasions at a 2-week interval and analyzed with Student’s *t* test for paired samples adopting an α-level of 0.05. The mean values calculated by combining the measurements of both operators were used for inter-group difference analysis. The technical error of measurement (TEM) was assessed with the formula [18],

$$ \mathrm{TEM}=\sqrt{\sum {d}_i^2/2n} $$

in which *d*_*i*_ was the difference between the first and second measurement on the *i*th sample and *n* was the whole sample number. As a result, all the measurements presented no significant difference according to the *t* test (*P* > 0.05). The technical error of measurement was 0.35°.

## Results

### Comparison of CA and LSA among different sagittal skeletal malocclusion groups (Table 3)

**Fig. 5:**
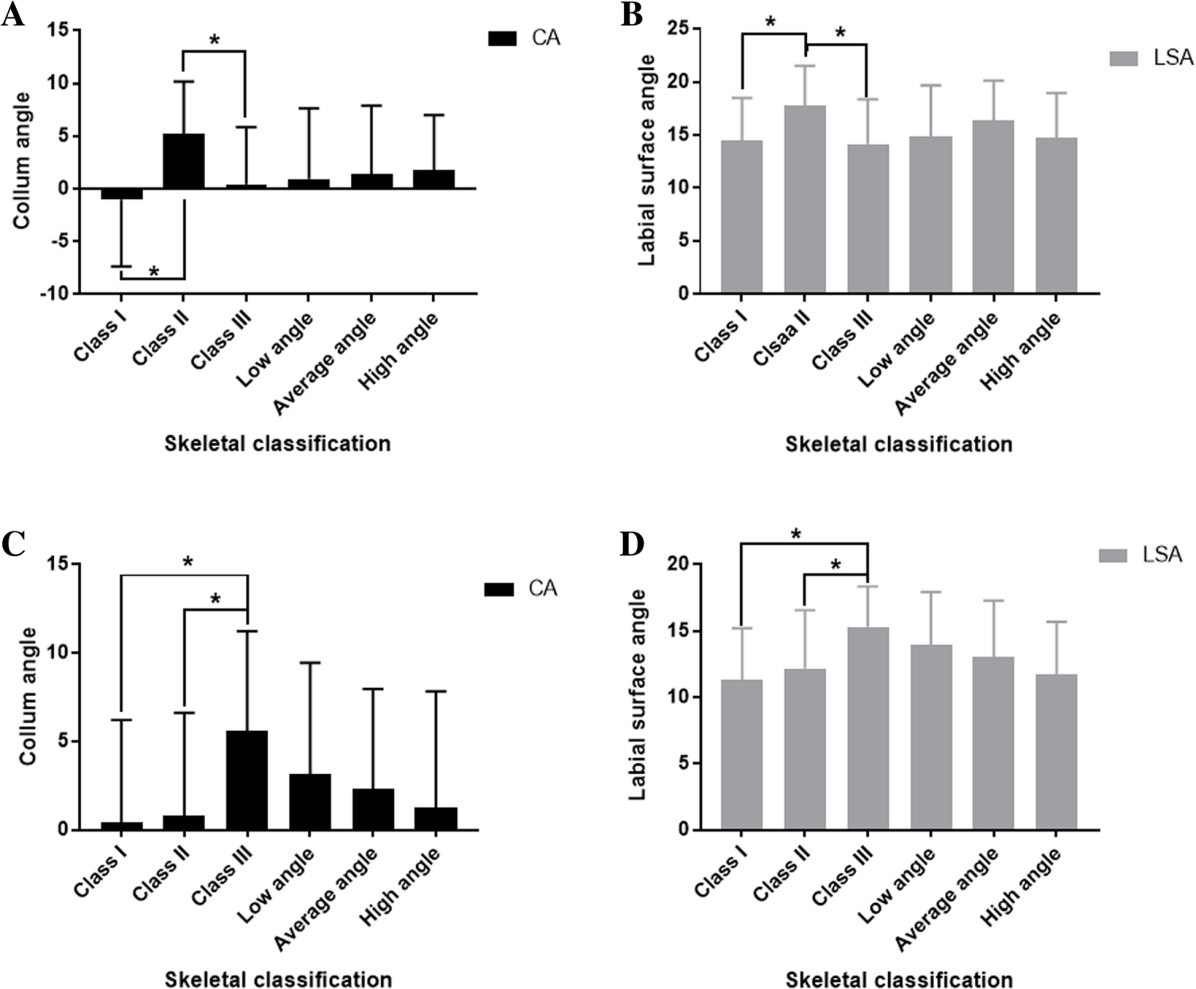
The value of CA and LSA in maxillary incisor of Class II (**a**, **b**) and mandibular incisor of Class III (**c**, **d**) are significantly greater than other groups. There is no statistical difference among different vertical skeletal classifications

In the maxilla, according to ANOVA, the mean values of CA in Class I, Class II, and Class III respectively achieved − 1.02 ± 6.30°, 5.18 ± 4.97°, and 0.43 ± 5.44°, and LSA were 14.44 ± 4.06°, 17.78 ± 3.74°, and 14.18 ± 4.20°. There were significant differences in both of the two measurements among different types of sagittal skeletal malocclusions (*P* = 0.002 < 0.01 and *P* = 0.008 < 0.01). Further Scheffe’s test was conducted for multiple comparisons. As a result, Class II patients had greater mean values of CA and LSA than patients in the other groups (I vs II: *P* = 0.003 < 0.01 and *P* = 0.028 < 0.05; II vs III: *P* = 0.030 < 0.05 and *P* = 0.019 < 0.05). No significant difference was noted between the Class I and Class III groups (*P* = 0.688 > 0.05 and *P* = 0.977 > 0.05) (Fig. 5a, b).

**Table 3:** Collum angle/labial surface angle of central incisors among different sagittal skeletal malocclusions (°)

	Class I		Class II		Class III		ANOVA *P* Value	Scheffe’s test		
	Number	Mean ± SD	Number	Mean ± SD	Number	Mean ± SD		I-II	I-III	II-III
Maxillary CA	24	−1.02 ± 6.30	20	5.18 ± 4.97	22	0.43 ± 5.44	0.002**	0.003**	0.688	0.030*
Maxillary LSA	24	14.44 ± 4.06	20	17.78 ± 3.74	22	14.18 ± 4.20	0.008**	0.028*	0.977	0.019*
Mandibular CA	24	0.40 ± 5.80	20	0.82 ± 5.78	22	5.59 ± 5.64	0.006**	0.970	0.013*	0.033*
Mandibular LSA	24	11.32 ± 3.91	20	12.18 ± 4.39	22	15.32 ± 3.05	0.002**	0.759	0.003**	0.034*

*P* < 0.05(*), *P* < 0.01(**)

In the mandible, the mean values of CA in Class I, Class II, and Class III were 0.40 ± 5.80°, 0.82 ± 5.78°, and 5.59 ± 5.64°, and LSA were 11.32 ± 3.91°, 12.18 ± 4.39°, and 15.32 ± 3.05°, respectively. Both the two measurements were also detected to be significantly different (*P* = 0.006 < 0.01 and *P* = 0.002 < 0.01). Furthermore, Class III groups had greater CA and LSA than the other two groups (I vs III: *P* = 0.013 < 0.05 and *P* = 0.003 < 0.01; II vs III: *P* = 0.033 < 0.05 and *P* = 0.034 < 0.05), while no difference was detected between Class I and Class II (*P* = 0.970 > 0.05 and *P* = 0.759 > 0.05) (Fig. 5c, d).

**Fig. 6:**
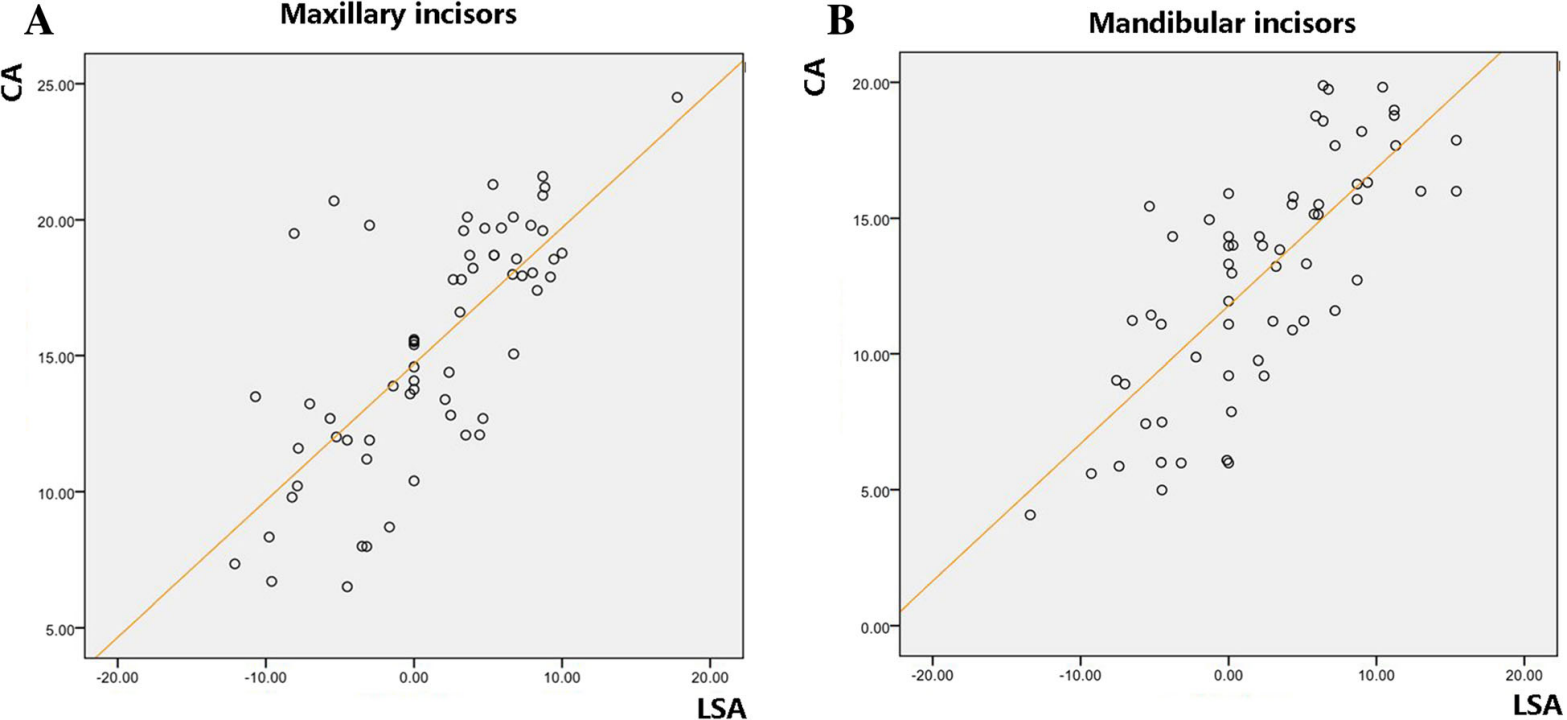
Both in the maxilla (**a**) and mandible (**b**), the CA and LSA are significantly and positively correlated

The consistency of the significant difference distribution implied us that there might be some extent correlation between the two measurements within the same jaw. Thus, we furtherly analyzed the association between CA and LSA within the same incisor by adapting the data from all the samples. As a result, the Pearson correlation test indicated that the CA and LSA were strongly positively correlated both in maxilla and mandible (upper jaw: *r* = 0.723, *P* = 0.000; lower jaw: *r* = 0.752, *P* = 0.000) (Fig. 6).

### Comparison of CA and LSA among different vertical skeletal malocclusion groups (Tables 4 and 5)

We detected no statistically significant differences in both CA and LSA among different vertical skeletal malocclusion groups (upper jaw: *P* = 0.915 > 0.05 and *P* = 0.347 > 0.05; lower jaw: *P* = 0.609 > 0.05; *P* = 0.217 > 0.05).

**Table 4:** Collum angle/labial surface angle of central incisors among different vertical skeletal malocclusions (°)

	Low angle		Average angle		High angle		ANOVA *P* value	Scheffe’s test		
	Number	Mean ± SD	Number	Mean ± SD	Number	Mean ± SD		L-A	L-H	A-H
Maxillary CA	21	0.94 ± 6.71	24	−1.02 ± 6.30	21	1.74 ± 5.28	0.915	0.974	0.916	0.981
Maxillary LSA	21	14.91 ± 4.76	24	14.44 ± 4.06	21	14.69 ± 4.29	0.347	0.503	0.986	0.416
Mandibular CA	21	3.13 ± 6.32	24	0.40 ± 5.80	21	1.25 ± 6.58	0.609	0.911	0.611	0.846
Mandibular LSA	21	13.94 ± 4.01	24	11.32 ± 3.91	21	11.73 ± 3.98	0.217	0.751	0.219	0.589

**Table 5:** Pearson correlation analysis indicated the significant positive correlation between CA and LSA (maxillary: *r* = 0.723, *P* = 0.000 < 0.001; mandibular: *r* = 0.752, *P* = 0.000 < 0.001)

	CA-LSA	
	*r*	*P*
Maxillary	0.723	0.000***
Mandibular	0.752	0.000***

*P* < 0.001(***)

## Discussion

The precise expression of anterior torque is essential to obtain normal overjet and overbite and achieve the satisfying esthetic effect and stable occlusal relationship. The ideal preadjusted torque in straight wire brackets is hard to accomplish adequately because of the material properties of wire, slot width, ligature selection, operation experience, individual tooth, and alveolar morphology [19]. Lots of studies found that the height and thickness of local alveolar predominantly restricted the range of anterior teeth movement [20], while less attention was paid to the limitation caused by the morphology. However, some orthodontists demonstrated that the variations in tooth morphology should be taken into deep consideration, which proved to be more important than the variations between the different types of preadjusted brackets [18].

The research about the influence of variability in incisor morphology on torque expression was first conducted by Bryant, who proposed three anatomic features of the maxillary central incisor [1]. The three features from a proximal view were the crown-root angulation (supplementary angle of the Collum angle) formed by the intersection of the longitudinal axis of the crown and the longitudinal axis of the root, the labial surface angle formed by a tangent to the bracket bonding point on the labial surface of the crown and the long axis of the crown, and the lingual curvature of the crown. The following morphological studies of anterior teeth mainly focused on the first two features [2, 19, 21].

Before the introduction of CBCT, visualization of the Collum angle and labial surface angle mainly depended on the lateral cephalogram, which might provide a magnified image with virtual distortion and controversial conclusion [5, 22–24]. The use of high-resolution CBCT enables us to measure the two anatomical features convincingly in three-dimension with quantitative and qualitative evaluating software [25]. Recently, researchers have used CBCT to examine the morphology of the anterior teeth, including the Collum angle and labial surface angle [2, 7]. Nevertheless, none of them investigated the differences among various skeletal malocclusions, even though the values of Collum angle of maxillary central incisors were found great differences among various Angle malocclusions [1, 5, 26, 27].

**Fig. 7:**
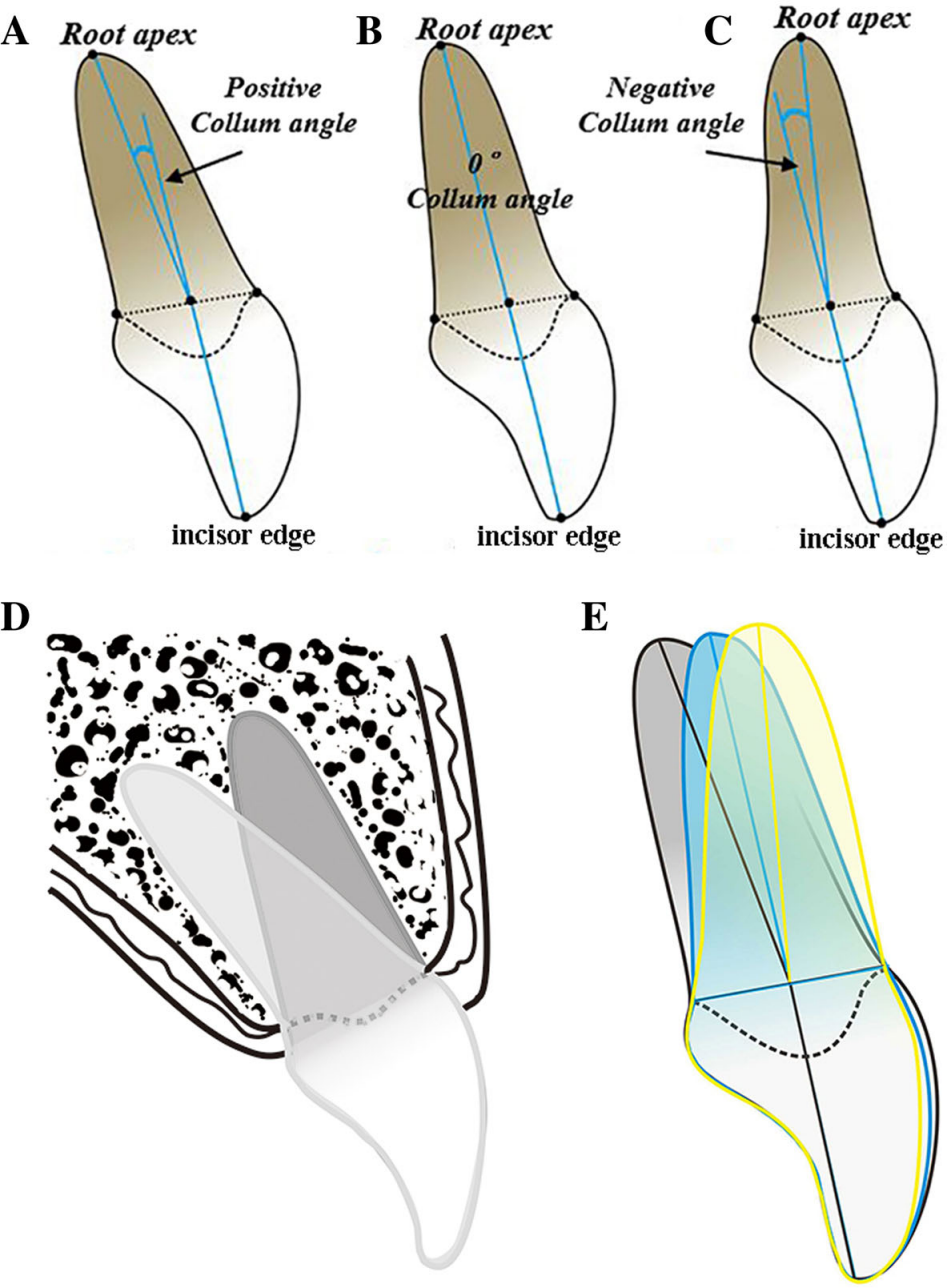
The various Collum angle in central incisor, the long axis of the root can deviate to the labial side (**a**) or lingual side (**c**) of the long axis of the crown, or coincidence (**b**). The schematic diagram indicates that the root bends toward lingual cortical alveolar because of Collum angle (**d**). The schematic diagram elucidates that the more obvious Collum angle accompanies with the greater labial surface curvature of the crown (**e**)

For the Collum angle (CA), our observation furtherly confirmed the widespread existence of the crown-root phenomenon, which was consistent with previous lateral cephalography studies [1, 4, 5, 18, 21, 26, 28, 29] (Fig. 7a–c). Generally, the morphology was susceptible during development, for the genetic and environmental factors, and the physiological mineralization of crown preceded that of root [12]. Thus, when erupting, forces from peroral muscles, mastication, and orthodontic appliance integrally changed the developmental direction or position [30, 31]. Previous studies had indicated that the CA differs among groups with different types of Angle malocclusion and notable lingual side bending of the long axis of crown relative to long axis of root in upper incisor in Angle Class II division 2 patient [1, 8, 10]. Hence, we hypothesized that the formation of CA might associate with facial growth pattern for the common environmental and genetic determinants. In addition, we excluded samples of Angle Class II division 2 because of the proved apparent CA in maxillary central incisor. In the current study, Class II (5.18 ± 4.97°) samples had significantly greater CA compared with Class I (− 1.02 ± 6.30°) and Class III (0.43 ± 5.44°) in maxillary, while in mandibular, the Class III (5.59 ± 5.64°) samples presented significantly greater CA compared with Class I (0.40 ± 5.80°) and Class II (0.82 ± 5.7°). Combining with previous viewpoints, we suggested that remarkable CA in the maxillary incisor of skeletal Class II and the mandibular incisor of Class III could cause the root to be closer to the lingual cortical alveolar compared with the other types of skeletal malocclusion, which increased the risk of dehiscence and fenestration, root resorption, and torque limitation in the process of labial inclination [1, 5, 10].

Labial surface angle (LSA) was another anatomical feature of the tooth, standing for the labial surface curvature of the crown [2]. Fredericks observed a variation of 21° when LSA measured at the point 4.2 mm apart from the incisor edge in the occlusal-gingival direction using 30 extracted incisors [1]. Thus, the individual variety of labial surface curvature led to elusive torque control on preadjusted appliances. Miethke indicated that there was considerable variation of labial surface curvature among teeth in different positions. The curvature of lower incisor was the smallest while the lower first molar was the largest, which was consistent with our results on LSA in maxillary and mandibular incisor (15.37 ± 4.27° vs 12.92 ± 4.14°). The significant discrepancy of LSA caused a wide range of torque 12.3 to 24.9° when detecting it at 4.5 mm apart from the occlusal surface [26]. Kong also found the value of LSA was significantly different at different heights from incisor edge, and the tangent point at a height from 3.5 to 5 mm, each 0.5 mm increase, the torque reduced by 1.5° [2]. Our study indicated that the values of LSA were greater in maxillary incisor of sagittal skeletal Class II malocclusion and mandibular incisor of Class III than other facial groups. Hence, when treating the same type of incisor with brackets with the same prefabricated torque at the same vertical height from the incisal edge, greater torque expression deviation might occur in the two groups of patients. Interestingly, our study also detected a significant positive correlation between the value of CA and LSA, meaning the labial surface curvature was correspondingly greater in cases with remarkable crown-root angulation. Hence, the root tip became easier to contact the lingual cortical alveolar and more challenging to avoid dehiscence and fenestration when labially inclined.

Consistent with the previous study, we detected no statistical difference in both CA and LSA among the vertical skeletal classifications. Harris found no correlation between CA and PP-FH, OP-FH, FH-MP, and lower face height ratio measurements standing for vertical growth pattern [5]. However, CA still affected the stress distribution of the periodontal ligament in the vertical direction with CA increasing and the center of tooth rotation gradually approached the dental cervix, which prevented the teeth from intruding into the alveolar bone [19, 32].

The cause of excessive lingual bending of incisor is still controversial at present, but more scholars prefer environmental factors. Harris reported that the mandibular incisor erupts earlier and provided restriction and guidance for the eruption of maxillary incisor when establishing occlusal contact. The remarkable CA of maxillary incisor usually accompanied by obvious anterior retroclination in Class III patients. In fact, these incisors presented excessive labial inclined feature due to compensatory reason. Moreover, other studies and present study found no significant difference compared with the Class I, so the conclusion of Harris was debatable [5]. Srinivasan furtherly discussed the relationship between the position of the lower lip line and CA and demonstrated that CA positive and increased when lower lip line ranged from the incisal 1/3 to middle 1/3, while the CA was negative and decreased when the lower lip line located at the crown cervix [8]. Mcintyre also agreed with the oral environmental contributors for the root tip 1/3 was still under mineralization after the eruption, which was sensitive to external forces [27]. Unlike the above views, Ruf and Pancherz reported no morphological difference in upper incisor between twins, one of whom belonged to Angle Class II division 1, another to Angle Class II division 2, even though with the higher located lower lip line [6], which illustrated the determinant role of genetic factors. Summing up the former viewpoints, we suggested that when the anterior occlusal relationship was initially established, neither the bite force conducting along the long axis of incisors was enough to resist tooth over eruption, nor balanced the perioral forces from tongue and lip. As a result, the crown-root angulation formed for the eruption direction of crown changed, while the root still mineralized along the assumptive pattern. Only when the incisor continued to erupt and balance with perioral muscle force, could the crown-root morphology be stabilized. Thus, it was important to coordinate oral and maxillofacial muscle function in preventing tooth abnormal morphology at the occlusion establishing stage.

## Conclusions

The maxillary incisor in sagittal skeletal Class II and mandibular incisor in Class III present greater crown-root angulation (Fig. 7d) and labial surface curvature than other types of malocclusion (Fig. 7e). There is a significant positive correlation between the two anatomical features. The above findings indicate that the morphologies of these teeth do play vital roles in torque variations, dehiscence, fenestration, and root resorption because of the root bending toward lingual cortical alveolar. Thus, when positioning a bracket, the variability of crown-root morphology is essential to be assessed before the operation.

## Abbreviations

ANB: A, A-point, deepest bony point on the contour of the premaxilla below ANS; B, B-point, deepest bony point on the contour of the mandible above pogonion; ANB, angle between point A, B and point N
ANOVA: One-way analysis of variance
CA: Collum angle
CBCT: Cone-beam computed tomography
CEJ: Cementoenamel junction
FHI: S-Go, the distance between lines parallel to Frankfurt plane passing through S and Go, represents the posterior facial height; N-Me, the distance between lines parallel to Frankfurt plane passing through N and Me, represents for the anterior facial height; FHI (S-Go/N-Me), facial height index, the ratio of posterior and anterior height, stands for vertical growth pattern of individual
FH-MP: The angle formed by the mandibular and Frankfurt plane, representing the extent of the vertical growth pattern
LSA: Labial surface angle
OP-FH: The angle formed by the occlusal and Frankfurt plane, representing the extent of the vertical growth pattern
PP-FH: The angle formed by the Palatal and Frankfurt Plane, representing the extent of the vertical growth pattern
SD: Standard deviation
SN-MP: S, sella, center of sella turcica; N, nasion, the most anterior limit of the frontonasal suture on the frontal bone in the facial midline; SN, connection between S and N, stands for anterior cranium base plane; Go, gonion, the most posterior inferior point of mandible angle; Me, menton, most inferior point of the bony chin; MP, connection between Me and Go, stands for mandibular plane; SN-MP, angle between SN and MP
SPSS software: Statistical Product and Service Solutions software
TEM: Technical error of measurement
Wits: The perpendicular lines are dropped from points A and B onto the occlusal plane, Wits is measured from Ao to Bo
